# Synergistic effects triggered by simultaneous Toll‐like receptor‐2 and ‐3 activation in human periodontal ligament stem cells

**DOI:** 10.1002/JPER.19-0005

**Published:** 2019-05-29

**Authors:** Alice Blufstein, Christian Behm, Johannes Gahn, Oksana Uitz, Ivana Naumovska, Andreas Moritz, Xiaohui Rausch‐Fan, Oleh Andrukhov

**Affiliations:** ^1^ Department of Conservative Dentistry and Periodontology University Clinic of Dentistry Medical University of Vienna Vienna Austria

**Keywords:** mesenchymal stem cells, periodontal ligament, Toll‐like receptor 2, Toll‐like receptor 3

## Abstract

**Background:**

Although periodontitis is associated with disruption of the host‐microbial homeostasis, viruses are currently discussed to influence disease progression. Viral pathogens are recognized by Toll‐like receptor (TLR)‐3, which engages a different signaling pathway than other TLRs. This study aimed to investigate the effect of TLR‐3 agonist polyinosinic:polycytidylic acid (Poly I:C) on the expression of inflammatory markers and bone metabolism proteins by human periodontal ligament stem cells (hPDLSCs) compared with TLR‐2 agonist Pam3CSK4, which mimics the effect of bacterial lipoproteins. To assess potential combined effects of bacterial and viral infections, hPDLSCs response to simultaneous TLR‐2 and TLR‐3 activation was investigated.

**Methods:**

HPDLSCs were stimulated with Poly I:C (0.0001‐1 µg/mL), Pam3CSK4 (1 µg/mL), and their combinations for 24 hours. Gene expression and protein levels of interleukin (IL)‐6, IL‐8, monocyte chemoattractant protein (MCP)‐1, and osteoprotegerin (OPG) were measured with qPCR and ELISA.

**Results:**

Production of IL‐6, IL‐8, MCP‐1, and OPG was significantly increased by Poly I:C or Pam3CSK4 to a similar extent. The levels of all inflammatory mediators induced by simultaneous stimulation with Poly I:C and Pam3CSK4 were significantly higher compared with single stimuli as well as to their summed response. Gene expression and protein levels of OPG were enhanced by Poly I:C, but by lesser extent than by Pam3CSK4. OPG levels upon simultaneous stimulation with Pam3CSK4 and Poly I:C were significantly lower compared with Pam3CSK4 stimulation alone.

**Conclusions:**

Simultaneous TLR‐2 and TLR‐3 activation synergistically triggers IL‐6, IL‐8, and MCP‐1 production, which was not observed for OPG. These findings suggest that TLR‐3 activation by viral infections might promote periodontitis progression.

## 1 INTRODUCTION

Periodontitis is a chronic inflammatory disease with multiple etiologic and contributory factors leading to destruction of periodontal soft and hard tissue.^1^ It is characterized by a bacterial overgrowth, leading to an impairment of the bacteria‐host homeostasis, which results in an excessive and inappropriate inflammatory response.^2^, ^3^ Recent studies suggest that viral infections such as herpesviruses possibly have some association with periodontitis, but their exact etiologic role is yet to be investigated.^4^, ^5^

Bacterial and viral pathogen‐associated molecular patterns (PAMPs) are recognized by the innate immune system via specific pattern recognition receptors, such as Toll‐like receptors (TLRs).^6^ Ten different TLRs have been discovered in humans so far, differing in their ligands, localization, structure, and partially in signaling pathways.^7^ Several members of the TLR family are supposed to be involved in the recognition of bacterial and viral components in periodontal disease.^8^ TLR‐2 is located on the cell surface and recognizes various PAMPs including lipoproteins, peptidoglycans, and lipoteichoic acids. As most of the other TLR family members, TLR‐2 uses an myeloid differentiation primary response 88 (MyD88)‐dependent signaling pathway.^9^, ^10^ In contrast, TLR‐3, which is found in the endosomal compartment and mainly responds to viral double‐stranded RNA, acts via an MyD88‐independent pathway.^7^ TLR‐4 is the only receptor activating both MyD88‐dependent and ‐independent pathway and is considered as an important sensor of bacterial lipopolysaccharide (LPS).^11^

TLRs play an important role in both periodontal health maintenance and periodontitis progression.^5^, ^8^ The contribution of TLR‐2 and TLR‐4 to periodontal disease is investigated at most. Clinical observations not only showed an enhancement of TLR‐2 and TLR‐4 expression in oral tissues during periodontitis, but also a crucial role of these receptors for periodontitis progression.^12^^‒^ 14 Interestingly, animal studies with different periodontitis models show that TLR‐2 deficient mice exhibit lower levels of alveolar bone loss and proinflammatory cytokine expression compared to wild‐type and TLR‐4‐deficient mice, which underlies the important role of TLR‐2 in periodontitis.^15^, ^16^ In contrast to TLR‐2 and TLR‐4, the role of TLR‐3 in periodontitis is much less investigated. TLR‐3 is thought to be involved in the recognition of different viral components and therefore the information about the effects of TLR‐3 in periodontal tissues might be important for understanding the role of viral infection in periodontitis progression.^17^ TLR‐3 activation is shown to induce inflammatory response in different cells of periodontal tissues, particularly in oral epithelial cells and gingival fibroblasts, but it remains largely unknown in human periodontal ligament stem cells (hPDLSCs).^5^ These fibroblast‐like cells reside in the perivascular space of the periodontium and fulfill the minimal criteria for mesenchymal stem cells (MSCs).^18^ In addition to differentiation capacity, hPDLSCs express different TLRs and contribute to inflammatory response.^19^, ^20^ These cells also exhibit immunomodulatory functions by either paracrine mechanisms or direct contact with immune cells.^18^, ^21^ Thus, hPDLSCs are not only involved in regulating the periodontal tissue homeostasis and regeneration, but potentially also in progression of periodontal disease.^18^, ^20^, ^22^

There is only limited knowledge about the influence of TLR‐3 activation in hPDLSCs. Stimulation with TLR‐3 agonist polyinosinic:polycytidylic acid (Poly I:C) leads to downregulation of the osteogenic capacity of hPDLSCs.^23^ Furthermore, a conference paper by Klincumhom et al. reported increased gene expression of immunomodulatory mediators interferon (IFN)‐γ and indoleamine‐pyrrole 2,3‐dioxygenase (IDO‐1) in hPDLSCs after TLR‐3 activation.^24^ Apart from IDO‐1, no study occupied with the effects of TLR‐3 activation on the expression of proinflammatory mediators by hPDLSCs.

The aim of the present in vitro study was to investigate the effects of TLR‐3 activation by Poly I:C in hPDLSCs on their inflammatory response and compare it to the already well investigated response triggered by TLR‐2 agonist Pam3CSK4.^25^^‒^ 27 The second aim was to assess the response of hPDLSCs to simultaneous stimulation with TLR‐2 agonist Pam3CSK4 and TLR‐3 agonist Poly I:C. Synergistic effects of simultaneous TLR‐2 and TLR‐3 activation have already been shown for enhancement of B‐cells, dendritic cells, and macrophages activation, but have never been reported in cells of periodontal tissues.^28^^‒^ 30 As target parameters, we have investigated the production of proinflammatory mediators interleukin (IL)‐6, IL‐8 and monocyte chemoattractant protein (MCP)‐1. The proinflammatory cytokine IL‐6 plays an important role in acute inflammation and induction of bone resorption.^31^ IL‐8 and MCP‐1 are chemoattractants promoting development of acute inflammation and inducing neutrophil and monocyte migration, respectively.^32^, ^33^ Furthermore, we investigated the expression of bone metabolism proteins osteoprotegerin (OPG) and receptor activator of NF‐κB ligand (RANKL), which are involved in the regulation of osteoclastogenesis and bone resorption.^34^

## 2 MATERIALS AND METHODS

### 2.1 Cell culture

The protocol for isolation of primary hPDLSCs was approved by the Ethics Committee of the Medical University of Vienna (ethical approval number: 1694/2015, revised in 2018). All experiments were performed according to the Declaration of Helsinki and the “Good Scientific Practice” guidelines of the Medical University of Vienna.

Primary hPDLSCs were obtained from third molars of 10 healthy individuals, which were extracted due to orthodontic indications. The donors (five female and five male) were white non‐smokers aged 18 to 22 years. All donors gave their informed written consent before tooth extraction. Isolation of hPDLSCs was performed as described previously and cultivated in Dulbecco's Modified Eagle's Medium (DMEM),^1^ supplemented with 10% fetal bovine serum (FBS)^2^ and 1% penicillin and streptomycin (P/S),^2^ in humid environment of 5% CO_2_ at 37°C.^25^ In the subsequent experiments, cells with passage levels 3 to 7 were used.

### 2.2 Confirmation of MSC character of hPDLSCs

Mesenchymal stem cell character was confirmed via analysis of MSC surface markers (CD29, CD73, CD90, CD105, CD146) and hematopoietic cell surface markers (CD31, CD34, CD45) by immunostaining and subsequent flow cytometry. In particular, cells were stained with the respective antibody conjugated with fluorophore. The following antibodies^3^ were used: fluorescein isothiocyanate (FITC)‐conjugated mouse anti‐human CD31, FITC‐conjugated mouse anti‐human CD34 and FITC‐conjugated mouse anti‐human CD45, phycoerythrin (PE)‐conjugated mouse anti‐human CD29, PE‐conjugated mouse anti‐human CD73, PE‐conjugated mouse anti‐human CD90, PE‐conjugated mouse anti‐human CD105 and PE‐conjugated mouse anti‐human CD146. Cells were analyzed using FACS device^4^ with an argon laser; acquisition was limited to 10,000 events.

### 2.3 3,4,5‐Dimethylthiazol‐2‐yl‐2,5‐diphenyl tetrazolium bromide (MTT) proliferation assay

hPDLSCs were seeded in 24 well plates at a density of 2 × 10^4^ cells per well in 0.5 mL DMEM supplemented with 1% P/S and 10% FBS. Following overnight incubation to allow adherence, cells were stimulated in triplicates with Poly I:C (0.0001‐1 µg/mL)^5^ in FBS‐free DMEM containing 1% P/S for 24, 48, or 72 hours. To analyze the cell proliferation/viability, 50 µL of 3,4,5‐dimethylthiazol‐2‐yl‐2,5‐diphenyl tetrazolium bromide (5 mg/mL) were added to each well and incubated for 2 hours. Afterwards, media were discarded, and cells were incubated with dimethylsulfoxide (300 µL/well) for 5 minutes. Absorbance was measured with a microplate reader^6^ at 570 nm.

### 2.4 Treatment protocol for gene and protein expression analysis

hPDLSCs were seeded in 24‐well plates at a density of 5 × 10^4^ cells per well in 0.5 mL DMEM supplemented with 1% P/S and 10% FBS and incubated overnight to allow adherence. Subsequently, cells were stimulated in triplicates with Poly I:C (0.0001‐1 µg/mL), Pam3CKS4 (1 µg/mL)^5^ or their combinations in FBS‐free DMEM containing 1% P/S for 24 hours, since we found in our preliminary experiments that the response after lower stimulation time (4 hours) is rather small (data not shown). The concentration of Pam3CSK4 was chosen based on our recent study.^35^ Untreated cells, cultivated only with FBS‐free DMEM supplemented with 1% P/S, served as control group.

### 2.5 Quantitative polymerase chain reaction

The gene expression levels of proinflammatory markers IL‐6, IL‐8, and MCP‐1, and bone metabolism proteins OPG and RANKL were measured with quantitative polymerase chain reaction (qPCR). Cell lysis, extraction of mRNA, reverse transcription into cDNA, and qPCR were performed using a commercially available kit,^7^ according to the manufacturer's instructions with a starting cell number of 5 × 10^4^. After conducting reverse transcription with a thermocycler,^8^ qPCR was performed.^9^ Gene Expression Assays^10^ with the following ID numbers were used: IL‐6, Hs00985639_m1; IL‐8, Hs00174103_m1; MCP‐1, Hs00234140_m1; OPG, Hs00900358_m1; RANKL, Hs00243522_m1; and GAPDH Hs99999905. The reactions were performed in duplicates and the cycle threshold (C_t_) was determined for each sample. Gene expression levels were quantified as n‐fold changes in the expression of target gene compared with unstimulated control using 2^−∆∆Ct^ method. ∆∆C_t_ was calculated by following formula: ∆∆C_t_ = (C_t_^target^ − C_t_^GAPDH^)_sample_ − (C_t_^target^ − C_t_^GAPDH^)_control_; n‐fold expression was calculated as 2^−∆∆Ct^. The housekeeping gene *GAPDH* was used as endogenous reference.

### 2.6 Enzyme‐linked immunosorbent assay

Following the stimulation protocol, conditioned media were collected and the protein concentration of IL‐6, IL‐8, and MCP‐1,^11^ as well as OPG and RANKL^12^ were analyzed with enzyme‐linked immunosorbent assay (ELISA). All kits were used according to the manufacturer's instructions and the samples were applied in duplicates. Optical densities were measured at 450 nm and plotted against a standard curve to determine protein concentrations.

### 2.7 Statistical analysis

Statistical analysis was performed using the statistical program SPSS 24.0.^13^ Experiments were conducted in triplicates for each donor and their mean values were calculated.

Statistical analysis was conducted with mean values obtained from cells of 10 different donors. Friedman test was applied to determine differences between groups, followed by Wilcoxon test for the pairwise comparison. Statistical significance was set at 0.05. Data are expressed as mean values ± standard error of the mean (SEM) of values obtained from 10 different donors.

## 3 RESULTS

### 3.1 Expression of MSC and hematopoietic surface markers in hPDLSCs

The results of the analysis of surface expression of specific mesenchymal and hematopoietic markers in hPDLSCs used in the present study are summarized in Table 1. hPDLSCs of all donors were stained positively (>96%) for all investigated MSC surface markers except CD146. The proportion of CD146+ cells comprised about 43% of all cells. Furthermore, hPDLSCs were negatively stained with all investigated hematopoietic markers (<2%).

**Table 1:** Mesenchymal and hematopoietic surface marker expression by hPDLSCs

MSC markers		Hematopoietic markers	
CD29	97.6 ± 0.2	CD14	2.0 ± 0.4
CD73	96.3 ± 0.2	CD31	0.8 ± 0.2
CD90	98.7 ± 0.3	CD42	0.8 ± 0.2
CD105	96.4 ± 0.4	CD45	1.8 ± 0.3
CD146	43.1 ± 6.3		

Expression of mesenchymal and hematopoietic surface markers by hPDLSCs was analyzed via immunostaining and subsequent flow cytometry. Data are presented as mean ± SEM from 10 hPDLSCs of 10 donors used in the present study.

### 3.2 Effect of different Poly I:C concentrations on cell proliferation/viability of hPDLSCs

**Figure 1:**
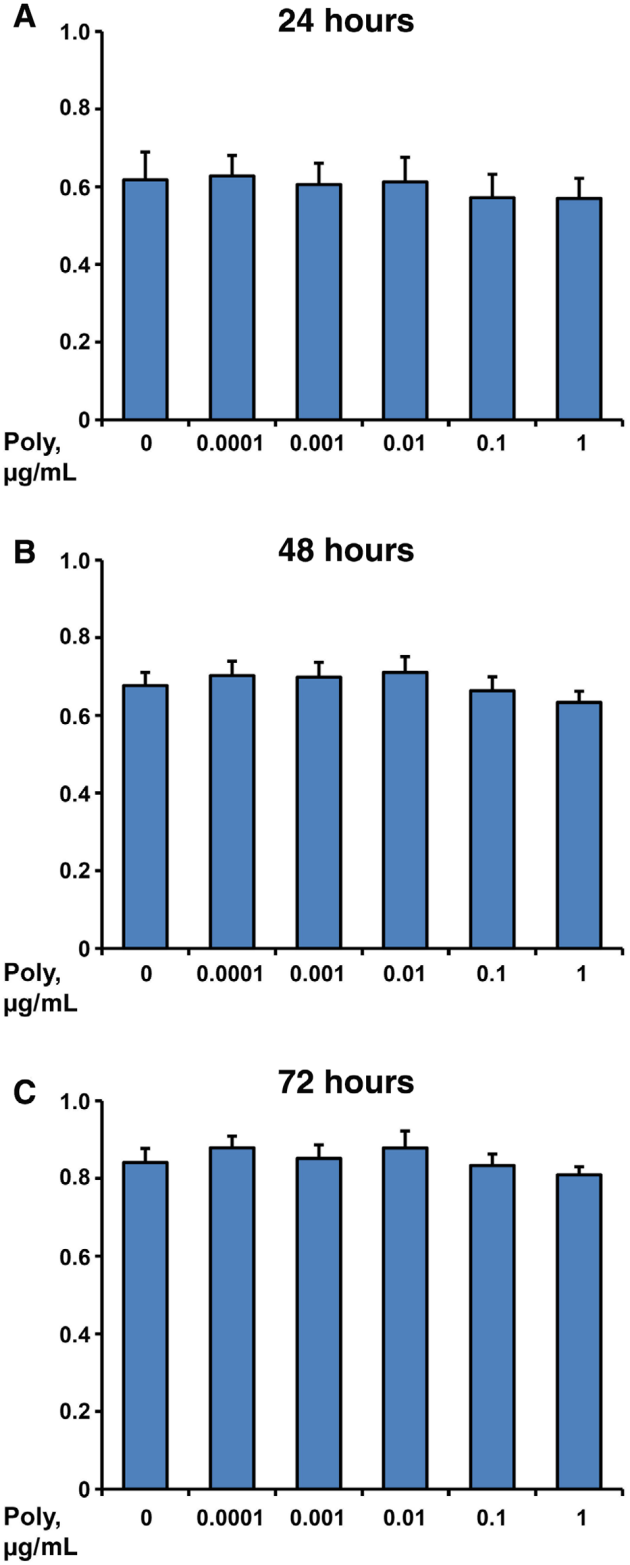
Effect of different Poly I:C concentrations on cell proliferation/viability of hPDLSCs. Primary hPDLSCs were stimulated with Poly I:C in concentrations from 0.0001 to 1 µg/mL and the cell proliferation/viability was assessed with MTT assay after 24 (**A**), 48 (**B**), and 72 hours (**C**). Y‐axes represent mean values ± SEM of optical densities measured at 570 nm in five independent experiments conducted on hPDLSCs from five different individuals

Cell proliferation/viability of hPDLSCs after stimulation with Poly I:C in concentrations of 0.0001 to 1 µg/mL for 24, 48, and 72 hours is shown in Figure 1; as shown, Poly I:C had no influence on cell proliferation/viability at all measured time points.

### 3.3 Effect of single and combined stimulation with Poly I:C and Pam3CSK4 on gene expression and protein levels of IL‐6 in hPDLSCs

**Figure 2:**
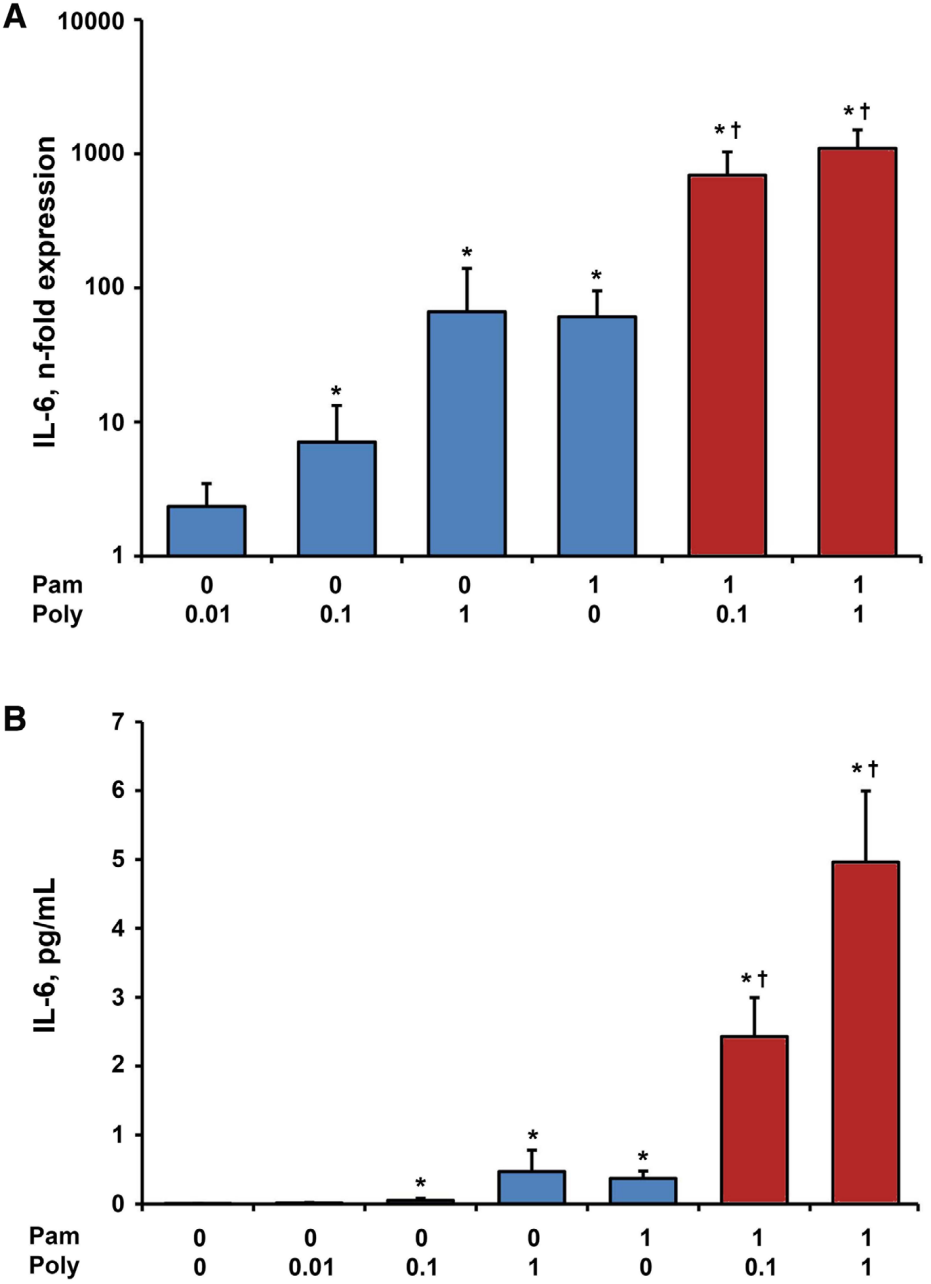
Effect of Poly I:C and/or Pam3CSK4 on gene expression and protein levels of IL‐6 in hPDLSCs. Primary hPDLSCs were stimulated with single TLR agonist (blue bars, concentrations are given in µg/ml) or their combinations (red bars) for 24 hours. Resulting gene expression levels of IL‐6 were measured with qPCR and are represented as n‐fold expression in relationship to untreated cells (**A**). Protein concentration of IL‐6 in the conditioned media (**B**) was analyzed with ELISA. All data are presented as mean ± SEM of 10 values obtained in independent experiments on hPDLSCs from 10 different healthy donors. ^*^Significantly higher versus control (untreated cells), *P* < 0.05. ^†^Significantly different between simultaneous stimulation and corresponding single stimulations, *P* < 0.05

Gene expression levels and protein concentrations of IL‐6 in response to stimulation with Poly I:C (0.01, 0.1, and 1 µg/mL), Pam3CSK4 (1 µg/mL) or their combinations are shown in Figure 2. Treatment with Poly I:C increased gene expression and protein levels of IL‐6 in a concentration dependent manner, which was statistically significant at concentrations of 0.1 and 1 µg/mL in comparison with untreated hPDLSCs. No difference between the responses of hPDLSCs to Poly I:C and Pam3CSK4 in concentrations of 1 µg/mL was observed. Simultaneous stimulation with Pam3CSK4 and 0.1 µg/mL Poly I:C resulted in about six‐fold higher IL‐6 protein levels compared with the sum of the responses induced by single stimuli. Furthermore, the protein level of IL‐6 in response to simultaneous stimulation with Pam3CSK4 and Poly I:C in a concentration of 1 µg/mL was about eight‐fold higher compared with the sum of protein levels after separate stimulation (see supplementary Figure 1 in online *Journal of Periodontology* for more details).

### 3.4 Effect of single and combined stimulation with Poly I:C and Pam3CSK4 on gene expression and protein levels of IL‐8 in hPDLSCs

**Figure 3:**
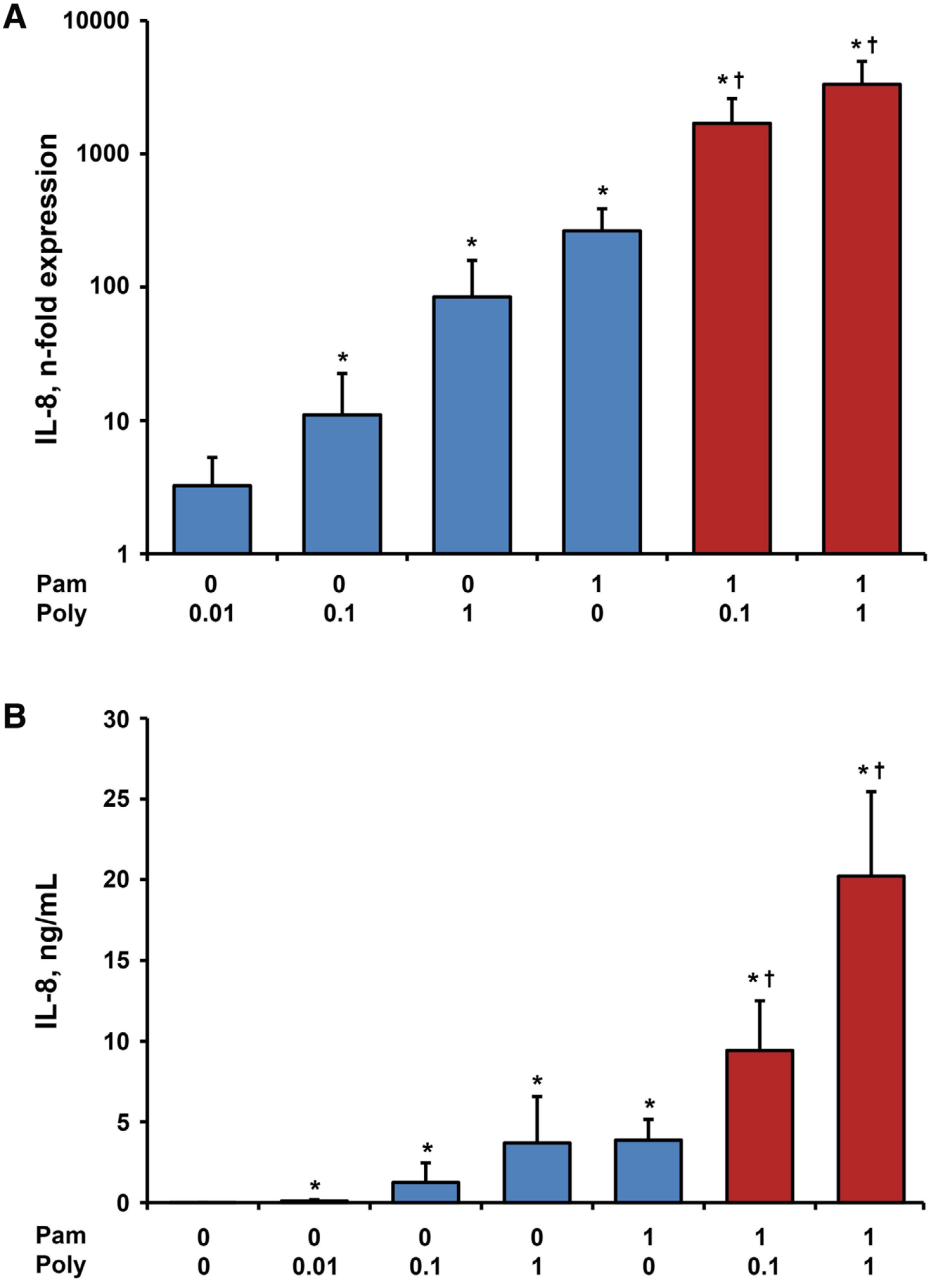
Effect of Poly I:C and/or Pam3CSK4 on gene expression and protein levels of IL‐8 in hPDLSCs. Primary hPDLSCs were stimulated with single TLR agonist (blue bars, concentrations are given in µg/ml) or their combinations (red bars) for 24 hours. Resulting gene expression levels of IL‐8 were measured with qPCR and are represented as n‐fold expression in relationship to untreated cells (**A**). Protein concentration of IL‐8 in the conditioned media (**B**) was analyzed with ELISA. All data are presented as mean ± SEM of 10 values obtained in independent experiments on hPDLSCs from 10 different healthy donors. ^*^Significantly higher versus control (untreated cells), *P* < 0.05. ^†^Significantly different between simultaneous stimulation and corresponding single stimulations, *P* < 0.05

Figure 3 shows the gene expression and protein levels of IL‐8 induced separate and simultaneous stimulation with Poly I:C (0.1 and 1 µg/mL) and Pam3CSK4 (1 µg/mL). Both, IL‐8 gene expression and protein concentration were enhanced by all tested Poly I:C concentrations in a dose‐dependent manner. No difference between the responses of hPDLSCs to Poly I:C and Pam3CSK4 in concentrations of 1 µg/mL was observed. Compared with the sum of separate stimulation with 0.1 µg/mL Poly I:C and Pam3CSK4, the protein concentration of IL‐8 was two‐fold higher in hPDLSCs treated with both agents simultaneously. Combined stimulation of 1 µg/mL Poly I:C with 1 µg/mL Pam3CSK4 resulted in a three‐fold higher protein concentration of IL‐8 in comparison to the summed response of single stimulations.

### 3.5 Effect of single and combined stimulation with Poly I:C and Pam3CSK4 on gene expression and protein levels of MCP‐1 in hPDLSCs

**Figure 4:**
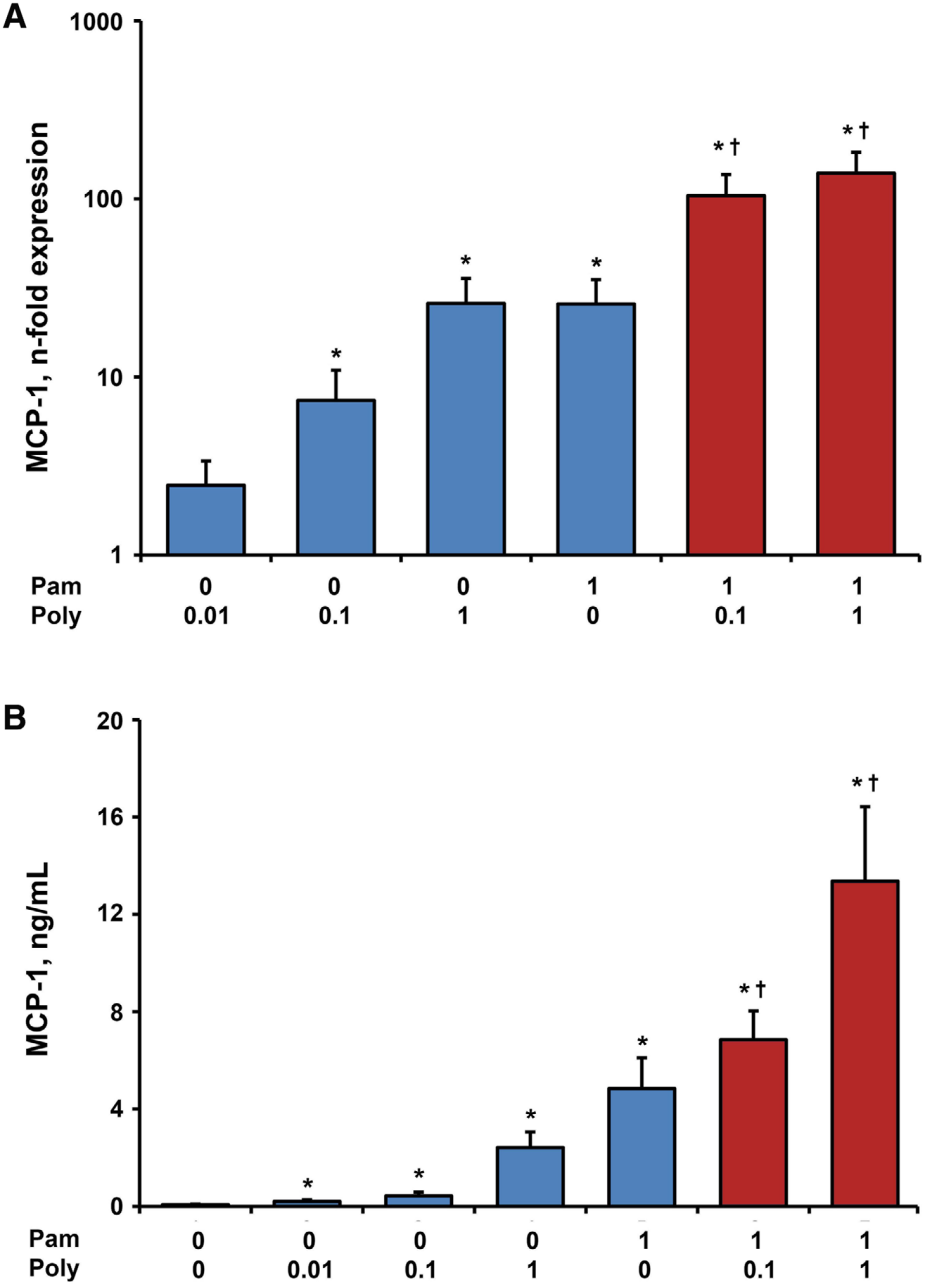
Effect of Poly I:C and/or Pam3CSK4 on gene expression and protein levels of MCP‐1 in hPDLSCs. Primary hPDLSCs were stimulated with single TLR agonist (blue bars, concentrations are given in µg/ml) or their combinations (red bars) for 24 hours. Resulting gene expression levels of MCP‐1 were measured with qPCR and are represented as n‐fold expression in relationship to untreated cells (**A**). Protein concentration of MCP‐1 in the conditioned media (**B**) was analyzed with ELISA and is represented in B. All data are presented as mean ± SEM of 10 values obtained in independent experiments on hPDLSCs from 10 different healthy donors. ^*^Significantly higher versus control (untreated cells), *P* < 0.05. ^†^Significantly different between simultaneous stimulation and corresponding single stimulations, *P* < 0.05

The effect of Poly I:C (0.1 and 1 µg/mL), Pam3CSK4 (1 µg/mL) and their combinations on the gene expression levels and protein concentration of MCP‐1 in hPDLSCs is presented in Figure 4. Similar to IL‐8, the gene expression and protein levels of MCP‐1 were increased by Poly I:C in a dose‐dependent manner. The response to single stimulation with 1 µg/mL Poly I:C was not statistically different compared with Pam3CSK4 at similar concentration. In comparison with the sum of responses induced by single stimuli, simultaneous stimulation of Pam3CSK4 and 1 µg/mL Poly I:C led to two‐fold higher protein levels of MCP‐1 (see supplementary Figure 1 in online *Journal of Periodontology*).

### 3.6 Effect of single and combined stimulation with Poly I:C and Pam3CSK4 expression of bone metabolism proteins by hPDLSCs

**Figure 5:**
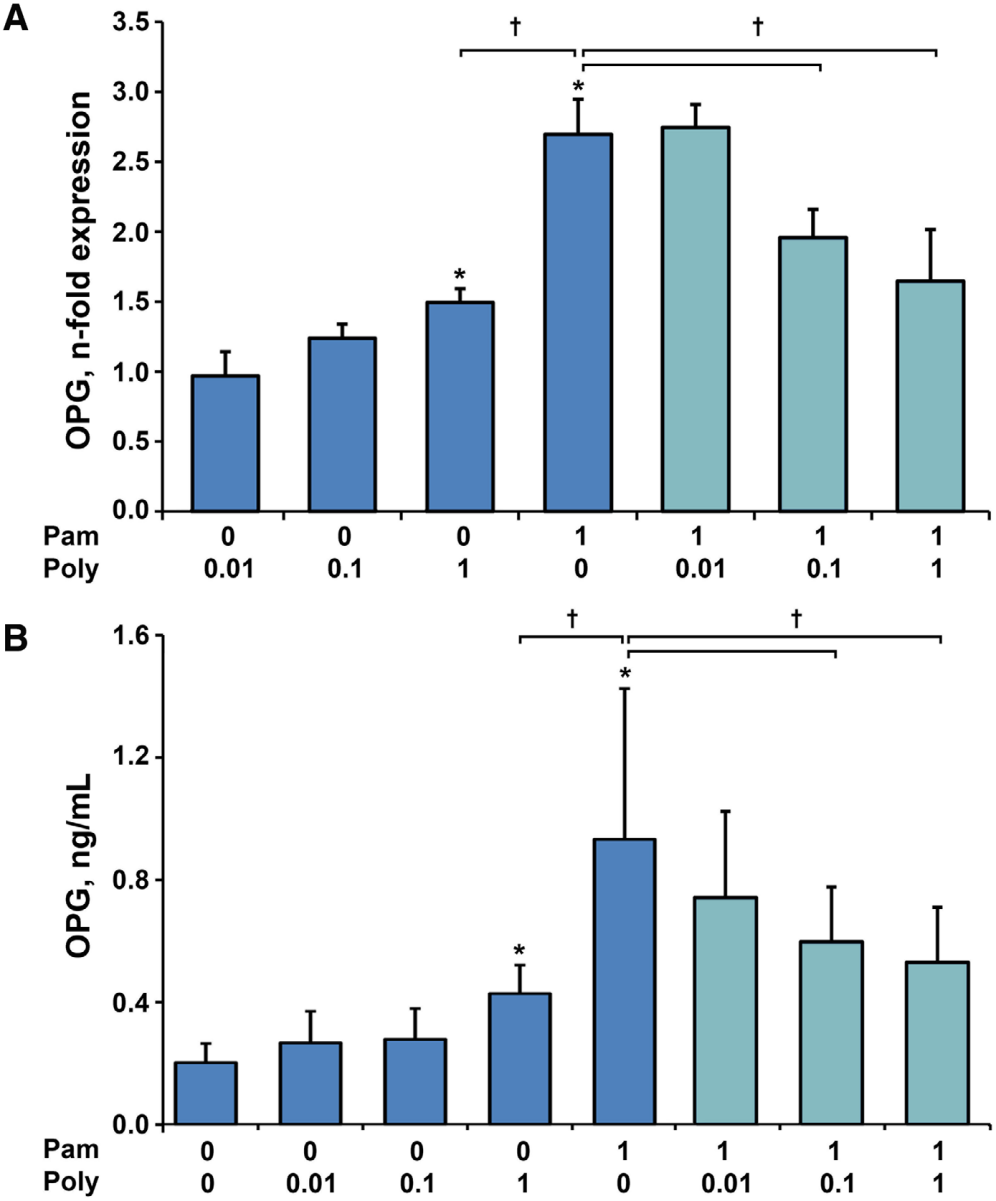
Gene expression levels of OPG in hPDLSCs and OPG protein concentration in conditioned media of hPDLSCs in response to either Poly I:C or Pam3CSK4 and in response to simultaneous stimulation. Primary hPDLSCs were stimulated with single TLR agonist (blue bars, concentrations are given in µg/ml) or their combinations (teal bars) for 24 hours. Resulting gene expression levels of OPG were measured with qPCR qPCR and are represented as n‐fold expression in relationship to untreated cells (**A**). Protein concentration of OPG in the conditioned media (**B**) was analyzed with ELISA. All data are presented as mean ± SEM of 10 values obtained in independent experiments on hPDLSCs from 10 different healthy donors. ^*^Significantly higher versus control (untreated cells), *P* < 0.05. ^†^Significantly different between simultaneous stimulation with Poly I:C and Pam3CSK4 and single stimulation with Pam3CSK4

The gene expression levels and protein concentration of OPG induced by stimulation of hPDLSCs with Poly I:C (0.01, 0.1, and 1 µg/mL), Pam3CSK4 (1 µg/mL) and their combinations are presented in Figure 5. Poly I:C led to a concentration dependent increase of OPG gene expression and protein level that was significant at the highest concentration. Stimulation with Pam3CSK4 also resulted in a statistically significant enhancement of OPG compared with untreated cells, which was significantly higher than by Poly I:C alone. Pam3CSK4‐induced OPG production was significantly decreased by combined stimulation with Poly I:C in concentrations of 0.1 and 1 µg/mL on both gene and protein level. In our experiments, RANKL was not detected on both gene expression and protein levels (data not shown).

## 4 DISCUSSION

The present in vitro study occupied with the effect of TLR‐3 agonist Poly I:C on the expression of inflammatory mediators and bone metabolism proteins by hPDLSCs. Furthermore, we investigated the effect of simultaneous stimulation of hPDLSCs with Poly I:C and TLR‐2 agonist Pam3CSK4 on the expression of different parameters. So, hPDLSCs obtained from 10 individuals were stimulated with various concentrations of Poly I:C either alone or in combination with Pam3CSK4 (1 µg/mL) for 24 hours.

We demonstrated that Poly I:C has no influence on the cell proliferation/viability of hPDLSCs. Furthermore, we investigated the effects of Poly I:C on the inflammatory response of hPDLSCs, particularly the gene expression and protein production of IL‐6, IL‐8, and MCP‐1, which are considered to have a crucial impact on periodontitis progression. We showed that Poly I:C leads to a concentration‐dependent increase of IL‐6, IL‐8, and MCP‐1 expression in hPDLSCs. The magnitude of the response induced by Poly I:C was comparable to that induced by TLR‐2 agonist Pam3CSK4. Hence, activation of TLR‐3 can promote inflammatory reactions and thus may play an important role in periodontitis progression.

An important observation of our study is that the production of proinflammatory mediators IL‐6, IL‐8, and MCP‐1 in response to simultaneous stimulation with TLR‐2 agonist Pam3SCK4 and TLR‐3 agonist Poly I:C was significantly higher than the sum of responses to stimulation with single substances. This was especially pronounced by the measurements of protein levels, which were up to eight‐fold higher in response to simultaneous stimulation compared with the sum of single stimuli. Thus, we can approve the synergism between the responses of hPDLSCs to TLR‐2 and TLR‐3 agonists regarding production of IL‐6, IL‐8, and MCP‐1. Until now, there are only few studies addressing the effects of TLR‐2 and TLR‐3 co‐activation in different host cells. Synergistic effects of simultaneous TLR‐2 and TLR‐3 activation have already been reported for enhancement of B‐cells, dendritic cells and macrophages activation.^28^^‒^ 30 Our study implies the existence of synergy between TLR‐2 and TLR‐3 responses also in hPDLSCs. In future studies, it would be important to prove the existence of such synergistic effect in other cells of periodontium, such as gingival fibroblasts (gingival MSCs) and oral epithelial cells. To date only one study reported that simultaneous stimulation of hGFs with Porphyromonas *gingivalis* LPS and Poly I:C for 12 to 24 hours does not lead to a significant increase of IL‐8 protein concentration in comparison to the sum of separate stimulation.^36^ Although commercial standard LPS preparations might activate both TLR‐2 and TLR‐4, the results of this study cannot be directly compared with our findings.^37^ Furthermore, this study used very high concentrations of Poly I:C (100 µg/mL) and *P. gingivalis* LPS (50 µg/mL), whereas we found synergistic effects at concentrations of 1 µg/mL for both Poly I:C and Pam3CSK4, which probably more appropriately resembles the concentration of these products in periodontal pocket.

The synergism between TLR‐2 and TLR‐3 induced responses in hPDLSCs might be explained by interactions between the intracellular signaling pathways activated by these proteins. TLR‐2 generally functions as heterodimer with either TLR‐1 or TLR‐6 and acts exclusively via an MyD88‐dependent pathway, whereas TLR‐3 acts exclusively through an MyD88‐independent pathway.^38^, ^39^ We can assume that the simultaneous activation of MyD88‐dependent and MyD88‐independent signaling by TLR‐2 and TLR‐3 agonists, respectively, results in synergistic response leading to extremely high cytokine production. TLR‐2 is usually activated by different bacterial lipoproteins, which are always present in periodontal pocket.^15^ Synthetic lipoprotein Pam3CSK4 leads to formation of a TLR2/1 heterodimer and resembles the activity of bacterial lipoproteins, whereas TLR‐3 is usually activated by viral DNA.^38^, ^40^ Thus, translating our finding to a clinical situation implies that the presence of viruses in dental biofilm might substantially enhance the host response to bacterial products. A synergism between TLR‐2 and TLR‐3‐induced response might promote inflammation and collateral tissue damages through attracting different immune cells and thus have an essential effect on disease progression. It is also hardly imaginable that the observed effects are specific only for Pam3CSK4, because in our recent study we showed that different TLR‐2 agonist induce qualitatively similar response in hPDLSCs.^35^

Interestingly, our previous studies show that the response of hPDLSCs to TLR‐4 agonist *E. coli* LPS is substantially (up to 10‐fold) lower compared with that of Pam3CSK4, although TLR‐4 receptors uses both MyD88‐dependent and MyD88‐independent signaling.^11^, ^25^, ^26^ This observation is quite surprising, because in the present study we showed that simultaneous activation of MyD88‐dependent and MyD88‐independent pathway leads to synergistic effects on IL‐6, IL‐8, and MCP‐1 production. It should be noted that activation of MyD88‐independent pathway through TLR‐3 and TLR‐4 is somewhat different. Both, TLR‐3 and TLR‐4 utilize the TRIF‐dependent signaling pathway. However, activation of TRIF by TLR‐4 is mediated by TRIF‐related adaptor molecule (TRAM), which is not implicated in TLR‐3 dependent signaling. Interestingly, a recent study on dendritic cells shows some differences in MyD88‐independent response induced by TLR‐3 and TLR‐4 agonists.^41^ Therefore, the exact pathways involved in TLR‐3 and TLR‐4 induced responses as well as their physiological significance still remain to be elucidated.

By considering potential clinical effects of simultaneous stimulation of hPDLSCs with TLR‐2 and TLR‐3 agonists it should be noted, that these cells produce several factors regulating the activity of the immune system. The parameters measured in our study usually promote inflammatory response through recruitment and activation of different immune cells. However, hPDLSCs possess strong immunosuppressive activity, which is mediated by the number of soluble factors as well as by direct cell‐to‐cell contact.^42^ TLRs are thought to play an important role in the immunomodulatory ability of different MSC‐like cells, but their exact role in the interaction of hPDLSCs is still to be clarified.^43^ Previous studies show that different TLR‐ligands activate several immunosuppressive proteins such as indoleamine‐2,3 dioxygenase, prostaglandin E2, protein death ligand 1.^42^ However, the contribution of proinflammatory and anti‐inflammatory activity of hPDLSCs into progression of periodontitis is still to be investigated.

Another aim of our study was the evaluation of bone metabolism proteins in response to Poly I:C, Pam3CSK4 and their combination. We focused on the gene expression and protein levels of RANKL, which is responsible for the regulation of osteoclast differentiation, and OPG, which functions as RANKL inhibitor.^34^ Gene expression and protein levels of OPG were significantly increased by Pam3CSK4 and Poly I:C alone. Based on our in vitro design, it is difficult to conclude if this increase in the OPG production could have a protective effect on the bone resorption in vivo. The clinical situation is very complex and bone metabolism proteins OPG and RANKL are produced by numerous cell types, including various immune cells.^44^ Simultaneous stimulation with TLR‐2 and TLR‐3 agonists led to a significant decrease of OPG production, compared with the stimulation with Pam3CSK4 alone. This observation is rather surprising and could be potentially explained by some autocrine mechanisms or the application of multiple inflammatory stimuli. As shown in our study, simultaneous stimulation of hPDLSCs by TLR‐2 and TLR‐3 agonists leads to drastically increased levels of proinflammatory mediators. OPG expression was reported to be enhanced by stimulation of hPDLSCs with proinflammatory cytokines IL‐1β and tumor necrosis factor‐α.^45^ However, this upregulation reached its peak at 12 hours and slightly decreased after 24 hours. The mechanisms underlying the observed effects of simultaneous activation of TLR‐2 and TLR‐3 on OPG production remain unclear and are yet to be investigated. Nevertheless, this inhibition might have a negative effect on bone turnover and promote bone resorption during periodontitis. RANKL was detected neither on gene expression, nor on protein level, which is in accordance with some other studies.^46^, ^47^ In contrast, some studies have measured RANKL expression in periodontal ligament cells as well as its regulation by TLR‐ligand and inflammatory cytokines.^48^, ^49^ The differences can be potentially explained by the cell source used in different studies. Particularly, a recent study demonstrates that RANKL expression is low in hPDLSCs isolated from healthy teeth and cells derived from resorbed teeth.^50^ Furthermore, high amounts of RANKL are produced by immune cells and therefore the control of cell purity is especially important.^44^ In our study, we worked with rather homogeneous population comprising of more than 96% of MSC‐like cells, whereas cell characterization was not always performed in other studies.

## 5 CONCLUSIONS

Although this study is limited by its in vitro character, the confrontation of cells with multiple stimuli allows a closer view into the clinical situation. According to our data, the presence of both bacterial and viral pathogens enhances the inflammatory response and inhibits production of bone metabolism proteins in hPDLSCs, which may contribute to periodontal tissue destruction. These synergistic effects by simultaneous activation with different TLR agonists might be a crucial factor in promoting progression of periodontal disease. More studies should occupy with this research topic to reveal the exact mechanisms underlying the interaction of different TLR signaling pathways and their role for periodontal heath and disease.

## Supporting information

Supplementary Figure 1. Protein concentration of IL‐6, IL‐8, and MCP‐1 in conditioned media of hPDLSCs after simultaneous stimulation versus sum of separate stimulations with Poly I:C and Pam3CSK4.

## Footnotes

1 Sigma‐Aldrich, St. Louis, MO.

2 Gibco, Carlsbad, CA.

3 eBiosciences, San Diego, CA.

4 Becton Dickinson, Franklin Lakes, NJ.

5 Invivogen, San Diego, CA.

6 Molecular Devices, Sunnyvale, CA.

7 TaqMan Gene Expression Cells‐to‐CT, Applied Biosystems, Foster City, CA.

8 Primus 96 advanced, PeqLab/VWR, Darmstadt, Germany.

9 ABI StepOnePlus, Applied Biosystems, Foster City, CA.

10 TaqMan Gene Expression Assays, Applied Biosystems, Foster City, CA.

11 Ready Set Go! Kits, ebioscience, Waltham, MA.

12 R&D Systems, Minneapolis, MN.

13 IBM, Armonk, NY.
