# Supplementary material for: Synergistic effects triggered by simultaneous Toll‐like receptor‐2 and ‐3 activation in human periodontal ligament stem cells
Source: J Periodontol. 2019 May 29;90(10):1190–201. doi: 10.1002/JPER.19-0005 (PMC6852053; doi:10.1002/JPER.19-0005)
Supplement: Supplementary file 1 — Supplementary Figure 1. Protein concentration of IL‐6, IL‐8, and MCP‐1 in conditioned media of hPDLSCs after simultaneous stimulation versus sum of separate stimulations with Poly I:C and Pam3CSK4. [file JPER-90-1190-s001.docx]

**Supplementary Figure 1**


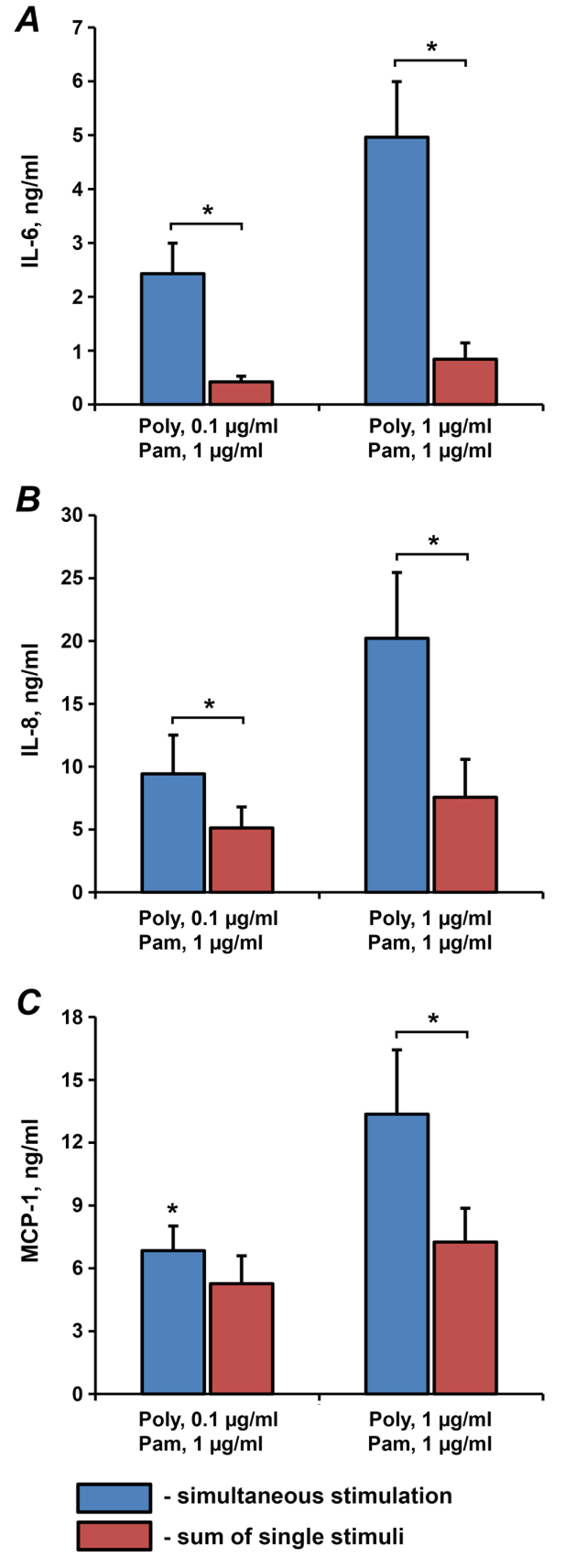


**Protein concentration of IL-6, IL-8 and MCP-1 in conditioned media of hPDLSCs after simultaneous stimulation vs. sum of separate stimulations with Poly I:C and Pam3CSK4.**

Primary hPDLSCs were stimulated with Poly I:C (0.1, 1µg/ml), Pam3CSK4 (1µg/ml) or their combinations for 24 hours and the protein concentration of IL-6 (A), IL-8 (B) and MCP-1 (C) in the conditioned media were measured with ELISA. Y-axes represent mean values ± S.E.M. of simultaneous stimulation vs. summed mean values ± S.E.M. of single stimulation with Poly I:C and Pam3CSK4 of 10 independent experiments conducted on hPDLSCs from 10 different individuals.

* - means significantly higher vs. stimulation with single agonist, p < 0.05

† - means significantly different between simultaneous stimulation and sum of single stimulation with Poly I:C and Pam3CSK4
